# Maternal Adiposity Influences Neonatal Brain Functional Connectivity

**DOI:** 10.3389/fnhum.2018.00514

**Published:** 2019-01-04

**Authors:** Andrew P. Salzwedel, Wei Gao, Aline Andres, Thomas M. Badger, Charles M. Glasier, Raghu H. Ramakrishnaiah, Amy C. Rowell, Xiawei Ou

**Affiliations:** ^1^Department of Biomedical Sciences and Imaging, Cedars-Sinai Medical Center, Biomedical Imaging Research Institute, Los Angeles, CA, United States; ^2^Department of Medicine, University of California, Los Angeles, Los Angeles, CA, United States; ^3^Department of Pediatrics, University of Arkansas for Medical Sciences, Little Rock, AR, United States; ^4^Arkansas Children’s Nutrition Center, Little Rock, AR, United States; ^5^Department of Radiology, University of Arkansas for Medical Sciences, Little Rock, AR, United States; ^6^Arkansas Children’s Research Institute, Little Rock, AR, United States

**Keywords:** brain development, functional connectivity, maternal obesity, neonatal offspring, resting-state fMRI

## Abstract

The neural mechanisms associated with obesity have been extensively studied, but the impact of maternal obesity on fetal and neonatal brain development remains poorly understood. In this study of full-term neonates, we aimed to detect potential neonatal functional connectivity alterations associated with maternal adiposity, quantified via body-mass-index (BMI) and body-fat-mass (BFM) percentage, based on seed-based and graph theoretical analysis using resting-state fMRI data. Our results revealed significant neonatal functional connectivity alterations in all four functional domains that are implicated in adult obesity: sensory cue processing, reward processing, cognitive control, and motor control. Moreover, some of the detected areas showing regional functional connectivity alterations also showed global degree and efficiency differences. These findings provide important clues to the potential neural basis for cognitive and mental health development in offspring of obese mothers and may lead to the derivation of imaging-based biomarkers for the early identification of risks for timely intervention.

## Introduction

In the United States, sixty percent of pregnant women are overweight and one third are obese (Kim et al., 2007; Moussa et al., 2016). Globally, more than 20% of women of reproductive age are estimated to be obese (Moussa et al., 2016). This represents a major public health concern since besides risks for obesity in the offspring, maternal obesity has also been associated with increased risk of adverse cognitive and neurobehavioral developmental outcomes in the next generation (Contu and Hawkes, 2017). However, the brain mechanisms underlying that remains largely unknown. The fetal and infancy periods foster the most dramatic brain development and are plastic and modifiable (Tau and Peterson, 2010; Gao et al., 2016), so a better understanding of the underlying neural basis during early brain development is essential for the development of potential intervention efforts (Gilmore et al., 2018). Among the limited existing studies, we have previously shown that maternal adiposity is associated with widespread reduction in white matter integrity (Ou et al., 2015) and altered functional connectivity within a prefrontal network at the neonatal stage (Li et al., 2016). Through imaging the offspring’s brain at the earliest time possible, these findings provide strong initial evidence of the *in utero* effects of maternal adiposity on offspring brain development.

In adolescents and adults with obesity, neuroimaging studies have documented widespread functional alterations in a variety of neural circuits that can be largely summarized in four main domains (Carnell et al., 2012); sensory cue processing (particularly visual ones) (Frankort et al., 2012); reward processing (Cohen et al., 2011; Shott et al., 2015); cognitive control (Frankort et al., 2012; Frank et al., 2013; Shott et al., 2015); and motor control (Rosenbaum et al., 2008). These four domains form a nexus that largely determines one’s feeding behavior from food sensing to reward evaluation, to executive monitoring/control of choices, and finally to eating behavior. For maternal obesity, it is possible that these functional alterations are transferred to the next generation through genetic and/or environmental pathways (Edlow et al., 2014) and lead to heightened odds not only for obesity, but also for other adverse cognitive, emotional, and behavioral outcomes given the wide range of functional circuits involved. Indeed, previous behavioral studies have shown the impact of maternal obesity on offspring cognition, emotion regulation, executive control, as well as internalizing and externalizing behaviors (Contu and Hawkes, 2017). Although our previous study focusing on the prefrontal network may provide a clue for part of the later cognitive differences that may be experienced by children born to mothers with higher adiposity, there are likely more widespread functional connectivity alterations that deserve further and more systematic exploration.

In this study, leveraging resting-state fMRI scans (Biswal et al., 1995) on 38 full-term neonates with maternal adiposity measures [i.e., body-mass-index (BMI) and body-fat-mass (BFM) percentages], we aimed to systematically examine whole-brain functional connectivity patterns to unveil the full spectrum of impact of maternal adiposity on the offspring’s brain. Specifically, based on a neonate-specific functional atlas (Shi et al., 2018), we segmented the brain images into 223 functionally more homogeneous units as seed regions and the potential maternal adiposity-related (normal vs. high BMI) alterations associated with each seed were examined. We hypothesized that there would be significant functional connectivity alterations among neural circuits associated with each of the four domains indicated in adult/adolescent obesity: sensory cue processing, reward processing, cognitive control, and motor control. After the seed-based studies, we further examined the global functional connectivity properties of each seed (i.e., global degree centrality and efficiency) (Rubinov and Sporns, 2010) to explore whether maternal adiposity also alters the whole brain functional connectivity pattern of each seed. Again, we hypothesized that multiple brain areas from each of the four hypothesized domains would show significant differences in global functional connectivity. Moreover, we also expected overlaps between the seed regions detected to show localized changes (i.e., seed-based findings) and those showing global connectivity alterations given the “nested” nature of the two examinations (i.e., voxel-wise whole brain functional connectivity patterns of one seed contribute to its nodal degree centrality and efficiency measures; Rubinov and Sporns, 2010). Finally, we hypothesized that all detected functional connections and global connectivity measures showing categorical differences between normal and high BMI groups would show significant correlations with BFM percentage of the mothers measured at early pregnancy.

## Materials and Methods

### Participants

Forty six pregnant women were originally enrolled for this study, which was approved by the local institutional review board. Informed consents were obtained from the pregnant mothers. Subjects were prospectively enrolled from a larger longitudinal study of pregnant women (ClinicalTrials.gov ID: NCT01131117). Inclusion criteria for the pregnant women were: pre-pregnancy self-reported BMI = 18.5–24.9 (normal BMI and weight) or BMI > 25 (high BMI; 14/15 subjects BMI ranged from 30 to 35, resulting in an obese classification); second parity, singleton pregnancy; ≥ 21 years of age; conceived without assisted fertility treatments. Exclusion criteria were: preexisting medical conditions; medical complications during pregnancy; medications during pregnancy known to influence fetal growth; smoking or alcohol drinking. All enrolled women had their body composition assessed using air displacement plethysmography (Bodpod, Cosmed, Chicago, IL, United States) and BMI measured within the first 10 weeks of gestation during their first research visit. Maternal IQ (MIQ) was assessed using the Wechsler Abbreviated Scale of Intelligence (WASI, Pearson, San Antonio, TX, United States), except for one subject who did not complete the IQ assessment. Total gestational weight gain was measured at 36 weeks of gestation. Birth weight and length of the infants were obtained and head circumference was measured at age 2 weeks. Only infants born full-term (≥37 weeks of gestation), with size at birth appropriate for gestational age (AGA), and without medical conditions known to influence growth and development were included in the study. In total, 44 infants participated in the MRI study, 43 had 3D T1 and RS-fMRI data prior to quality control, but 5 of them were later excluded due to excessive motion during the RS-fMRI using criteria specified in the data analysis section. The remaining 23 infants born to normal-BMI mothers and 15 infants born to mothers with high BMI were included in the data analysis.

### Imaging

At ∼2 weeks of age, MRI examination of the brain was conducted in the Department of Radiology at the Arkansas Children’s Hospital. Infants were fed 15∼30 min prior to the scan, swaddled in warm sheets, and immobilized using a MedVac Infant Immobilizer (CFI Medical Solutions, Fenton, MI, United States). No sedation was used. A pulse oximeter probe (InVivo Corp, FL, United States) was placed on a foot to monitor oxygen saturation and heart rate, and mini-muffs and a headset were placed over the ears to protect the infants from the noise generated during the scan. The MRI examinations were performed on a 1.5 Tesla Achieva MRI scanner (Philips Healthcare, Best, the Netherlands) with 60 cm bore size, 33 mT/m gradient amplitude, and 100 mT/m/ms maximum slew rate. A pediatric 8-channel SENSE head coil was used. A neonatal brain MRI protocol was used, which included sagittal 3D T1 weighted reconstructed to 3 planes, axial T2 weighted, axial diffusion weighted, and axial susceptibility weighted imaging sequences. This conventional neonatal MRI protocol was used for the investigators to potentially exclude subjects with apparent brain abnormalities. The 3D T1 weighted images acquired at 1 × 1× 1 m^3^ resolution were also used in the subsequent data analysis for imaging registration. In addition, a single-shot gradient echo T2^∗^-weighted EPI sequence with TR/TE 2400 ms/50 ms, acquisition voxel size 2 × 2 × 4 mm^3^ reconstructed to 1.251 × 1.251 × 4 mm^3^ and 150 volumes (scan duration of 6 min) was used to acquire the RS-fMRI data.

### Image Preprocessing

Functional data were preprocessed using the FMRIB Software Library (FSL v5.0.8; Jenkinson et al., 2012) and the Analysis of Functional NeuroImages suite (AFNI v16.0.10 2-25-2016; Cox, 1996). Steps included discarding initial volumes, motion correction, spatial smoothing (FWHM = 6 mm), motion censoring (Power et al., 2012) [i.e., frame-wise displacement (FD) > 0.3 and < 5 continuous volumes], linear interpolation of censored time-points, and bandpass filtering (0.01–0.08 Hz). Confound regression was used to reduce motion contamination based on recent guidelines (Ciric et al., 2017). Specifically, the confound regression strategy consisted of motion scrubbing plus a 36-parameter nuisance signal model; 9 regressors [i.e., eroded white matter (WM), eroded cerebral spinal fluid (CSF), gray mater (GM), and six parameters corresponding to rigid-body motion correction], their derivatives, quadratic terms, and squares of derivatives. Nuisance regression was performed via linear regression. Here, censored time points were ignored, and the output time-series was normalized. Five subjects were excluded based on excessive motion (i.e., >approximately 33% of volumes scrubbed). The total number of volumes (or degrees of freedom) was controlled across subjects using the participant with the fewest number of volumes post-scrubbing (*n* = 102 time-points). Excess volumes were discarded starting from the end of the functional time-series. There was no significant difference in mean FD between infants born to normal BMI (n-BMI, mean = 0.26, standard deviation = 0.40) and high BMI (h-BMI, mean = 0.29, standard deviation = 0.37) mothers; *t*(36) = -0.27, *p* = 0.789.

### Registration

Spatial normalization was achieved using a combination of within- and between-subject transformations. The UNC neonate template was used for co-registration (i.e., standard space) (Shi et al., 2011). Specifically, within-subject functional-to-anatomical alignment was achieved using rigid-body registration (FSL flirt). Note the inverse of this transformation was used to align the tissue-specific (i.e., WM, CSF, GM) masks for confound signal extraction. Importantly, image contrast was low/mixed in the anatomical images thus we used a combination of tools that we’ve found improves brain extraction or “skull-stripping” in the neonatal brain (FSL-bet plus the AFNI script @NoisySkullStrip). Between subjects anatomical-to-standard alignment was achieved using the advanced normalization tools (ANTS) suite (Avants et al., 2011), which consisted of rigid-body, affine, and diffeomorphic warping. Finally, functional-to-anatomical and anatomical-to-standard transformations were combined and then applied to the functional data. The functional data were finally resliced to 3 × 3 × 3 mm^3^ resolution with additional smoothing (FWHM = 8.0 mm).

### Functional Connectivity (FC) Analyses and Related Statistics

We used a neonatal derived functional connectivity (FC)-based atlas (UNC-CEDARS INFANT) (Shi et al., 2018) to generate seed-based FC measures (Biswal et al., 1995). For each sub-region defined in the atlas (*n* = 223; see Supplement A for correspondence with automated anatomical labeling (AAL) atlas), Fishers’ Z-transformed whole brain FC maps, defined as the temporal correlation between mean time course for the seed region and each voxel in the brain, were used to compute voxel-wise differences between groups (two-sample unpaired *t*-tests; n-BMI vs. h-BMI). Here, multiple comparisons were accounted for using a nonparametric permutation approach; FSL randomize (P-corrected < 0.05): 5000 permutations with threshold-free cluster enhancement (TFCE) (Smith and Nichols, 2009). Clusters were further summarized using center-of-mass (CM) coordinates and approximate AAL location based on spatial overlap. For each detected cluster we performed an additional *post hoc* analysis using the average cluster-level FC for each subject. Moreover, the relationship between cluster-level measures and other participant characteristics (e.g., infant’s race and maternal BFM) were assed using analysis of variance (ANOVA) and correlation-based analyses. The subject with missing IQ was excluded in the covariate analysis of that variable. Finally, correlation matrices were constructed and used to generate complimentary graph theoretical measures. Specifically, we used the graph theoretical network analysis toolbox (GRETNA) (Wang et al., 2015) to compute nodal degree and efficiency for each seed region. Nodal degree describes the information communication ability of the node within the network and is defined as the number of links connected to the node. Nodal efficiency characterizes the efficiency of parallel information transfer of the node at the local level and is defined as the average of the inverse shortest path length computed at the neighborhood of the node. The correlation matrices were positive-binarized and thresholded across subjects using the sparsity metric (0.05–0.5 in 0.05 increments). The sparsity metric makes use of subject-specific connectivity strength thresholds in order to match the ratio of actual edges-to-maximum possible edges in the network, ensuring the same number of edges for each network and thus allowing for an examination of the relative network organization. Nodal degree and efficiency measures were computed for each sparsity increment with the area-under-curve (AUC) scalar serving as input to subsequent analyses. Mirroring the seed-based cluster analysis, we tested for differences in nodal degree/efficiency between groups and calculated their relationship with respect to other participant characteristics. Similarly, global measures, including network or global efficiency and small-worldness, were also compared between the two groups. Global efficiency measures the global efficiency of parallel information transfer in a network. The local efficiency of the network measures how efficient communication is among the first neighbors of a given node when it is removed. Small-world networks have a shorter characteristic path length than regular networks (high clustering and long path lengths) but greater local interconnectivity than random networks (low clustering coefficient and short path lengths). The small-world metric supports both specialized/modularized and integrated/distributed information processing and maximizes the efficiency of information transfer at a relatively low wiring cost.

## Results

### Participant Characteristics

The demographic information for the infants (and their mothers) included in the data analysis is listed in Table 1. The two groups of infants were not different in gender, race, birth weight and length, weight/length/age/head circumference at the time of MRI, gestational age, or maternal IQ. The two groups were significantly different in maternal body-mass-index (BMI) and BFM percentage, as required by study design. Gestational weight gain was also different between the two groups, in line with the US Institute of Medicine weight gain recommendations.

**Table 1:** Demographic information (mean ± standard deviation) for the neonates born to mothers with normal or high body-mass-index (BMI).

	Normal BMI n-BMI (*N* = 23)	High BMI h-BMI (*N* = 15)	*P-*value
Gender (M/F)	12/11	8/7	1
Race (Caucasian/others)	17/6	11/4	1
Birth weight (kg)	3.42 ± 0.48	3.65 ± 0.55	0.17
Birth length (cm)	50.63 ± 2.68	50.67 ± 2.63	0.98
Weight at MRI (kg)	3.60 ± 0.44	3.81 ± 0.44	0.34
Height at MRI (cm)	51.02 ± 2.01	51.30 ± 1.77	0.93
Head circumference at MRI (cm)	36.07 ± 1.22	36.06 ± 0.93	0.62
Age at MRI (days)	14.35 ± 1.70	14.13 ± 1.64	0.75
Gestational age (days)	275.65 ± 7.06	274.33 ± 6.33	0.67
Maternal IQ	106.68 ± 10.17	107.67 ± 8.31	0.86
Maternal body-mass-index (BMI)	22.20 ± 1.75	32.81 ± 2.29	<0.001
Maternal body-fat-mass (BFM) percentage	28.56 ± 4.61	43.72 ± 4.96	<0.001
Gestational weight gain (kg)	12.68 ± 2.16	9.04 ± 4.30	<0.001

### Maternal Adiposity and Neonate Functional Connectivity

**FIGURE 1:**
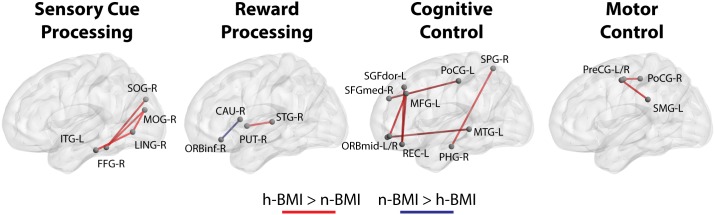
Functional connections that show significant differences (see section Materials and Methods) between neonates born from normal BMI (n-BMI) mothers and those from mothers with high BMI (h-BMI), categorized in four domains, including visual cure processing, reward processing, cognitive control, and motor control. Red connections: h-BMI > n-BMI. Blue connections: n-BMI > h-BMI. BrainNet Viewer (Xia et al., 2013, http://www.nitrc.org/projects/bnv/).

**FIGURE 2:**
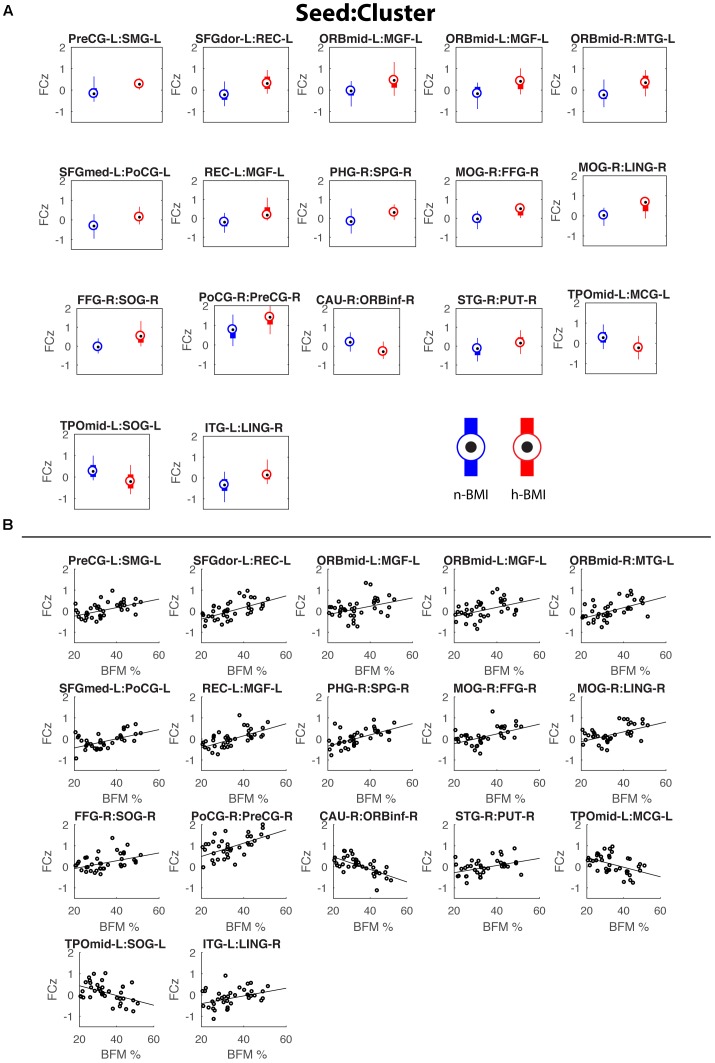
Box plots showing the functional connectivity differences (in z scores) between the two groups for the 17 detected connections **(A)** and corresponding scatter plots for the correlations between functional connectivity and maternal fat mass percentage **(B)**. See Table 2 for details of statistics.

Our seed-based analysis detected 17 clusters associated with 15 seed sub-regions showing significant functional connectivity differences between neonates born to mothers with normal (n-) or high (h-) BMI; Table 2 – Statistical Summary, Figure 1 – Connection Visualization, and Figure 2A – Group Distributions. *Post hoc* cluster-level analyses indicated that 15 out of 17 clusters showed significant group differences after correcting for the 223 seed regions (Bonferroni correction: *p* < 0.05/223 or 0.0002). Among the 17 clusters, 14 demonstrated hyper-connectivity in the high BMI group (h-BMI > n-BMI). Importantly, the connections covered all four hypothesized domains of functional circuits involved in the etiology of adult obesity with a particularly dense coverage on cognitive control areas, including the lateral prefrontal and orbital frontal cortices. Specifically, for sensory cue processing, four vision-related connections were detected to show hyper-connectivity in the h-BMI group (relationships denoted as seed: cluster using AAL nomenclature); right fusiform gyrus to right superior occipital (FFG-R:SOG-R), right middle occipital gyrus to FFG-R (MOG-R:FFG-R), MOG-R to right lingual gyrus (MOG-R:LING-R), and left inferior temporal gyrus to LING-R (ITG-L:LING-R). For reward processing, one connection between right superior temporal gyrus and right putamen (STG-R:PUT-R) was h-BMI hyper-connective while another between right caudate and right inferior orbital frontal (CAU-R:ORBinf-R) was detected to show h-BMI hypo-connectivity (i.e., n-BMI > h-BMI). For cognitive control, seven connections were detected to show hyper-connectivity in the h-BMI group; the left rectus gyrus to left medial frontal gyrus (REC-L:MGF-L), two separate connections involving the left middle orbital frontal gyrus and MFG-L (ORBmid-L:MFG-L), ORBmid-R to left middle temporal gyrus (ORBmid-R:MTG-L), the right parahippocampul gyrus to right superior parietal gyrus (PHG-R:SPG-R), left dorsal superior frontal gyrus to REC-L (SFGdor-L:REC-L), and the left medial superior frontal gyrus to left post-central gyrus (SFGmed-L:PoCG-L). For motor control, two connections were detected with h-BMI related hyper connectivity; one between PoCG-R and right precentral gyrus (PoCG-R:PreCG-R) and another involving the PreCG-L and left supramarginal gyrus (PreCG-L:SMG-L). Finally, there were two additional connections associated with the left middle temporal pole and left middle cingulate cortex (TPOmid-L:MCG-L) or left superior occipital cortex (TPOmid-L:SOG-L) that showed h-BMI hypo-connectivity. All the detected connections showing functional connectivity strength differences between the two groups also correlated with individual measures of adiposity (i.e., maternal body fat mass (BFM) percentage, see Table 2 and Figure 2B).

**Table 2:** Summary of seed-based brain functional connectivity results for neonates born to normal or high BMI mothers (see Supplement A for abbreviations of the AAL regions).

Seed		Cluster					n-BMI (*N* = 23)		h-BMI (*N* = 15)		n-BMI vs. h-BMI		BFM %	
funPar #	AAL	AAL	# Vox	CM RL	CM AP	CM IS	μ	*SE*	μ	*SE*	*t*	*p*	r	p
1	PreCG-L	SMG-L	2	39.5	23.0	23.5	-0.10	0.06	0.30	0.07	-4.26	**0.0001**	0.51	0.0009
14	SFGdor-L	REC-L	11	1.0	-26.2	-12.0	-0.18	0.07	0.34	0.09	-4.79	**0.0000**	0.59	**0.0001**
41	ORBmid-L	MGF-L	29	24.0	-23.6	18.2	-0.09	0.07	0.45	0.11	-4.39	**0.0001**	0.39	0.0165
41	ORBmid-L	MGF-L	28	26.4	-7.0	31.4	-0.14	0.07	0.37	0.09	-4.46	**0.0001**	0.45	0.0051
42	ORBmid-R	MTG-L	26	34.0	24.0	-3.6	-0.19	0.07	0.35	0.09	-4.74	**0.0000**	0.54	0.0005
66	SFGmed-L	PoCG-L	6	40.5	5.5	19.0	-0.29	0.06	0.17	0.07	-5.29	**0.0000**	0.55	0.0004
72	REC-L	MGF-L	47	13.7	-0.6	36.1	-0.23	0.06	0.33	0.09	-5.72	**0.0000**	0.63	**0.0000**
106	PHG-R	SPG-R	24	-15.6	56.6	29.1	-0.14	0.07	0.34	0.07	-4.58	**0.0001**	0.60	**0.0001**
128	MOG-R	FFG-R	28	-16.7	40.5	-6.9	-0.11	0.05	0.45	0.09	-5.85	**0.0000**	0.52	0.0008
129	MOG-R	LING-R	15	-15.3	40.9	-5.9	0.00	0.05	0.54	0.09	-5.49	**0.0000**	0.52	0.0007
139	FFG-R	SOG-R	54	-16.2	58.0	20.0	-0.01	0.05	0.49	0.10	-5.14	**0.0000**	0.43	0.0070
146	PoCG-R	PreCG-R	2	-25.0	15.5	38.5	0.70	0.08	1.33	0.10	-4.82	**0.0000**	0.56	0.0002
174	CAU-R	ORBinf-R	5	-10.3	-10.3	-9.5	0.25	0.06	-0.29	0.09	5.42	**0.0000**	-0.66	**0.0000**
192	STG-R	PUT-R	6	-17.5	11.0	5.5	-0.20	0.06	0.22	0.08	-4.13	0.0002	0.41	0.0113
207	TPOmid-L	MCG-L	43	-0.6	32.4	27.5	0.31	0.07	-0.21	0.09	4.45	**0.0001**	-0.46	0.0035
207	TPOmid-L	SOG-L	4	17.0	47.0	26.5	0.29	0.07	-0.20	0.11	3.91	0.0004	-0.45	0.0043
212	ITG-L	LING-R	14	-13.0	59.6	-6.9	-0.33	0.08	0.15	0.08	-4.22	**0.0002**	0.38	0.0179

*Bold values indicate significance at P < 0.0002 (Bonferroni corrected; 0.05/223) funPar #, the sub-region number of each detected seed in our reference neonatal functional atlas; # Vox, number of voxels within the detected clusters; CM RL/AP/IS, MNI coordinates of the detected clusters in right-left, anterior-posterior, inferior-superior directions; n-BMI, normal body-mass-index group; h-BMI, high BMI group. BFM, maternal body-fat-mass percentage; μ, mean; SE, standard deviation; t, t-value; p-p value. r, Pearson correlation value.*

**FIGURE 3:**
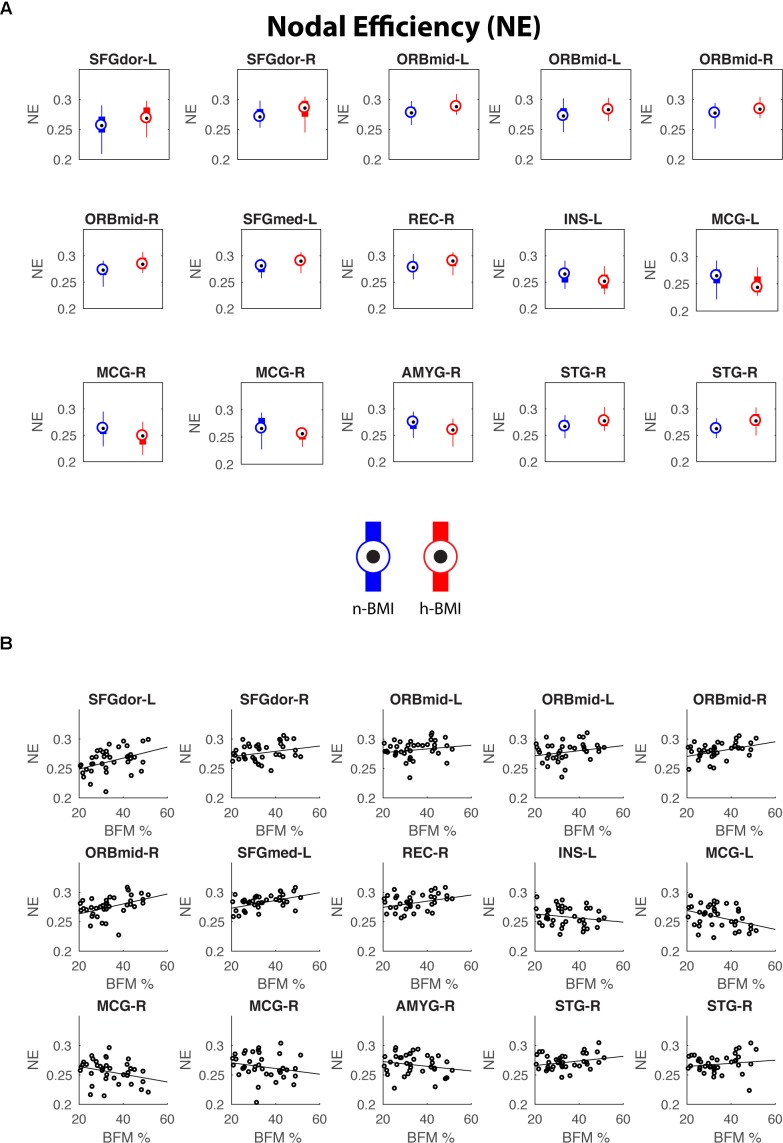
Regions showing significant nodal degree centrality differences between the two groups **(A)** and their correlations with maternal fat mass percentage **(B)**. See Table 3 for details of statistics.

**FIGURE 4:**
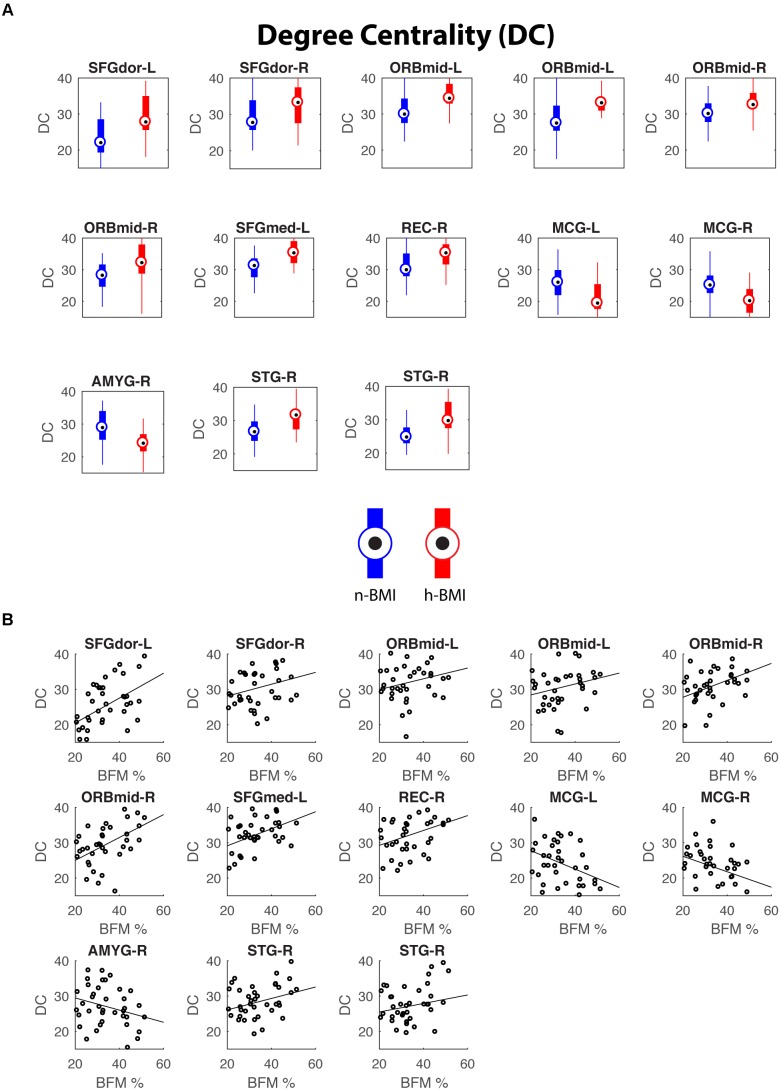
Regions showing significant nodal efficiency differences between the two groups **(A)** and their correlations with maternal fat mass percentage **(B).** See Table 4 for details of statistics.

Our graph theoretical analysis revealed two sets of regions showing nodal degree centrality and/or nodal efficiency differences between the n-BMI and h-BMI groups (*p* < 0.05 uncorrected); Table 3 and Figure 3 – Nodal Degree Centrality; Table 4 and Figure 4 –Nodal Efficiency. Specifically, for nodal degree, 13 sub-regions were detected, including six sub-regions associated with reward processing [i.e., two sub-regions in each of the bilateral middle orbital frontal cortices (ORBmid-L/R), one sub-region in the right amygdala (AMYG-R), and another one in the right rectus (REC-R)], three sub-regions related to cognitive control [i.e., one sub-region in each of the bilateral dorsal superior frontal gyrus (SFGdor-L/R), and the other one in the left medial superior frontal gyrus (SFGmed-L)], and four other sub-regions [i.e., one sub-region in each of the bilateral middle cingulate cortex (MCG-L/R) and two sub-regions in right superior temporal gyrus (STG-R)]. All regions showing significant degree differences also showed significant differences in nodal efficiency. Only one sub-region in the left insula (INS-L) and another one in MCG-R showed lower efficiency in the h-BMI group without showing significant group differences in degree, indicating a high level of overlap between the nodal degree and efficiency measures. More importantly, three of the functional sub-regions detected to show significant local connectivity differences (i.e., SFGdor-L; ORBmid-L, and ORBmid-R, Figure 1) also showed significant differences in nodal degree and nodal efficiency. If examined at the whole AAL region level, five regions, including SFGdor-L, ORBmid-L/R, SFGmed-L, and STG-R, consistently showed significant differences in both local connectivity strength and graph measures of nodal degree and efficiency. Although to a lesser degree, most of the regions showing nodal degree and/or efficiency differences also demonstrated correlations between corresponding degree/efficiency measures and maternal fat mass percentage (Tables 3, 4 and Figures 3B, 4B). Finally, we did not detect any significant group differences in global efficiency or small-worldness.

**Table 3:** Summary of graph measure results – node degree centrality for neonates born to normal or high BMI mothers.

Node		n-BMI (*N* = 23)		h-BMI (*N* = 15)		n-BMI vs. h-BMI		BFM %	
funPar #	AAL	μ	*SE*	μ	SE	*t*	*p*	*r*	*p*
14	SFGdor-L	23.38	1.21	29.04	1.59	-2.88	0.007	0.48	0.002
19	SFGdor-R	29.00	1.03	32.89	1.53	-2.19	0.035	0.26	0.109
39	ORBmid-L	30.27	1.11	35.07	1.15	-2.89	0.006	0.25	0.136
41	ORBmid-L	28.83	1.25	33.51	1.22	-2.55	0.015	0.23	0.164
42	ORBmid-R	29.84	1.01	33.39	1.14	-2.29	0.028	0.43	0.007
44	ORBmid-R	27.85	1.02	32.59	1.71	-2.54	0.016	0.48	0.003
62	SFGmed-L	30.81	0.85	35.43	0.99	-3.50	0.001	0.47	0.003
79	REC-R	30.71	1.10	34.80	1.29	-2.38	0.023	0.34	0.037
92	MCG-L	25.63	1.14	21.37	1.39	2.35	0.024	-0.40	0.013
95	MCG-R	24.85	1.04	20.10	1.29	2.87	0.007	-0.35	0.029
109	AMYG-R	28.72	1.17	24.26	1.19	2.57	0.015	-0.27	0.100
188	STG-R	26.87	0.85	30.87	1.12	-2.87	0.007	0.31	0.056
190	STG-R	25.59	0.79	29.81	1.78	-2.45	0.019	0.19	0.246

*funPar #, the sub-region number of each detected seed in our reference neonatal functional atlas; n-BMI, normal body-mass-index group; h-BMI, high BMI group. μ, mean; SE, standard deviation; t, t-value; p-p value. BFM, maternal body-fat-mass percentage; r-Pearson correlation value.*

**Table 4:** Summary of graph measure results – nodal efficiency for neonates born to normal weight or high BMI mothers.

Node		n-BMI (*N* = 23)		h-BMI (*N* = 15)		n-BMI vs. h-BMI		BFM %	
funPar #	AAL	μ	*SE*	μ	SE	*t*	*p*	*r*	*p*
14	SFGdor-L	0.26	0.004	0.27	0.005	-2.36	0.024	0.41	0.010
19	SFGdor-R	0.27	0.003	0.28	0.004	-2.05	0.048	0.27	0.099
39	ORBmid-L	0.28	0.003	0.29	0.003	-2.44	0.020	0.19	0.249
41	ORBmid-L	0.27	0.003	0.29	0.003	-2.57	0.014	0.24	0.145
42	ORBmid-R	0.28	0.003	0.29	0.003	-2.22	0.033	0.41	0.010
44	ORBmid-R	0.27	0.003	0.28	0.005	-2.15	0.038	0.43	0.007
62	SFGmed-L	0.28	0.002	0.29	0.003	-3.50	0.001	0.49	0.002
79	REC-R	0.28	0.003	0.29	0.003	-2.34	0.025	0.34	0.038
84	INS-L	0.26	0.003	0.25	0.004	2.13	0.040	-0.19	0.253
92	MCG-L	0.26	0.004	0.25	0.005	2.29	0.028	-0.37	0.021
95	MCG-R	0.26	0.004	0.25	0.005	2.61	0.013	-0.30	0.064
96	MCG-R	0.27	0.003	0.25	0.006	2.17	0.037	-0.21	0.212
109	AMYG-R	0.27	0.004	0.26	0.004	2.13	0.040	-0.21	0.210
188	STG-R	0.27	0.002	0.28	0.003	-2.39	0.022	0.27	0.104
190	STG-R	0.26	0.002	0.27	0.005	-2.18	0.036	0.15	0.363

*funPar #, the sub-region number of each detected seed in our reference neonatal functional atlas; n-BMI, normal body-mass-index group; h-BMI, high BMI group. μ, mean; SE, standard deviation; t, t-value; p-p value. BFM, maternal body-fat-mass percentage; r-Pearson correlation value.*

When we assessed the relationships between the observed functional connectivity alterations and other participant characteristics, only gestational weight gain showed widespread associations (*p* < 0.05) with the cluster-level functional connectivity measures (Supplement B). The other participant characteristics demonstrated minimal relationships with all three connectivity measures.

## Discussion

In this study, we detected significant functional connectivity alterations in a cohort of neonates born to mothers with high BMI (14/15 obese) compared to those born to mothers with normal BMI. The nineteen detected functional connectivity alterations cover all four hypothesized domains that are critically implied in adolescent/adult obesity mechanisms, including sensory cue processing (particularly visual ones), reward processing, cognitive control, and motor output control. Importantly, when examined at whole brain level, three of the nineteen regions involved in these alterations showing local functional connectivity changes also demonstrated significant global degree and efficacy differences. This overlap suggests that there are likely more widespread local connectivity changes associated with these regions that are statistically undetectable based on the current sample size but their collective effects cascade to produce significant global connectivity property changes as measured by global nodal degree and efficiency. Finally, all the functional connections and most of the nodal degree/efficiency measures detected to show significant group differences also quantitatively correlated with measures of maternal adiposity.

Little work has been done in humans to investigate the genetic and/or environmental mechanisms by which maternal obesity influences offspring cognition and mental health (Edlow et al., 2014). However, it is estimated that the heritability of the obesity trait is between 40 and 70% making it one of the strongest genetically influenced traits of humans (Willyard, 2014). In animal models, decreased proliferation of neuronal progenitor cells and abnormal synaptic activity, alterations of brain-derived neurotrophic factor and Notch signaling genes, and disrupted dendrite branching have all been reported in animal offspring born to obese mothers (Tozuka et al., 2009; Yu et al., 2014; Hatanaka et al., 2017), providing a certain structural basis for potential functional circuitry alterations. In this study, given the reported multi-domain functional deficits associated with adult obesity, including sensory cue processing, reward processing, cognitive control, and motor control (Carnell et al., 2012), we speculate that these traits are likely transferred to the offspring through genetic and/or environmental pathways leading to the high heritability of the obesity trait and related cognitive/mental phenotypes across generations. Indeed, we detected functional connectivity alterations in neonatal offspring of high BMI mothers in all four hypothesized domains, providing a strong neural basis for the across-generation transmission of maternal adiposity related outcomes.

Within the sensory cue processing domain, four connections were detected to show differences in functional connectivity between the two groups, including right middle occipital cortex-right fusiform, right middle occipital cortex-right lingual gyrus, right fusiform-right superior occipital cortex, and left inferior temporal gyrus-right lingual gyrus. Note three of the four connections are within the classical visual cortices and one is between the visual cortex and the neighboring inferior temporal gyrus that is part of the ventral visual pathway. This pattern, together with the fact that all four connections show hyper-connectivity in the high BMI group, may suggest stronger interactions within visual processing circuits, which may underlie the heightened sensitivity to visual food cues associated with obesity (Doolan et al., 2014). Consistent with our findings, adult studies have shown greater fMRI activation in visual cortices when processing high-calorie food stimuli among people with obesity, including inferior/middle occipital cortex and fusiform gyrus (Frankort et al., 2012; Nummenmaa et al., 2012).

Reward processing has been a major topic of obesity studies for decades (Carnell et al., 2012; Stice et al., 2013). In human reward circuitry, the ventral tegmental area, the striatum including both the caudate and putamen, the amygdala, the insula, the hippocampus and parahippocampus, the rectus, and the orbital frontal cortex, are all critical nodes mediating the brain’s reward evaluation of external stimuli (Haber and Knutson, 2010). Lying in the center of potential brain mechanisms for obesity, disrupted functional activation within and functional connectivity between these regions were frequently reported in adult/adolescent obesity studies (Cohen et al., 2011; Frankort et al., 2012; Frank et al., 2013; Shott et al., 2015). In the current study, two functional alterations-between the right caudate and right inferior orbital frontal cortex; and between the right superior temporal gyrus and right putamen were observed in neonatal offspring of mothers with high BMI. Moreover, global degree/efficiency changes were also detected for the middle orbital frontal cortex, the rectus, the insula, and the amygdala, indicating wide-spread disruption of functional interactions associated with reward-processing regions in the offspring of mothers with high BMI. Our findings of changes in reward-related functional connectivity in neonatal offspring of mothers with high BMI suggest that such reward-circuits alterations are likely programmed early-on and can be observed as early as in neonates.

Perhaps the most salient finding in this study relates to the dominant detection of functional connectivity differences in the cognitive control domain. Specifically, among the nineteen connectivity alterations detected, seven are associated with cognitive control of reward between classical cognitive control regions (i.e., prefrontal, temporal, and parietal regions) and reward evaluation/processing regions (i.e., regions within the orbital frontal cortex, the rectus, and the parahippocampal cortex). Among the cognitive control regions involved, five are from the prefrontal regions, one is from the left middle temporal gyrus, and one is from the left superior parietal gyrus. All three prefrontal regions in which obesity-associated differences were observed, including the left dorsal superior frontal gyrus, left middle frontal gyrus, and left medial superior frontal gyrus, are critical executive control regions frequently implied in functional deficits associated with reward re-evaluation, conflict monitoring, and choice selection among obese subjects (Cohen et al., 2011; Frankort et al., 2012; Frank et al., 2013). Importantly, all three prefrontal regions detected to show local functional connectivity differences also demonstrated global functional connectivity differences when measured using both degree and efficiency measures, again indicating the likely more widespread but sub-threshold functional connectivity alterations associated with these areas which may collectively contribute to the global connectivity differences detected. These results, together with our previous observation of functional connectivity changes within a prefrontal network identified using independent component analysis (ICA) in offspring born to obese mothers, converge to suggest the presence of significant alterations of functional circuits associated with cognitive control in offspring of mothers with adiposity during pregnancy. In addition to prefrontal regions, the middle temporal gyrus has been linked to emotional food memories and consistent with our findings, significant functional connectivity abnormalities of this area has been previously reported in a cohort of adolescents with obesity (Moreno-Lopez et al., 2016). Finally, the superior parietal gyrus represents an essential node in the brain’s dorsal attention network that largely governs attentional allocation to external stimuli. Consistent with our findings, aberrant functional connectivity of the superior parietal lobule has been previously documented in obese adults (Geha et al., 2017). Overall, there is a clear left hemisphere bias in the cognitive control regions detected in this study showing differences in functional connectivity with reward processing regions, which is in line with the left hemisphere bias in the adult functional brain topology related to cognitive control.

In addition to functional connections related to visual cue processing, reward processing, and cognitive control, we also detected one connection between the right precentral and right postcentral gyrus showing hyper-connectivity in the high BMI group compared with the normal weight group. This connection is clearly related to motor control and in theory might contribute to disrupted eating behavior. Finally, four other connections associated with the right superior temporal gyrus and the left temporal pole regions were also detected. Consistently, previous findings have shown reduced brain activation to food logos in superior temporal regions in obese compared with normal weight children (Bruce et al., 2013). A correlation between body mass measures and local blood flow in the temporal pole region in retired obese athletes was also observed (Willeumier et al., 2012). These findings implied that additional functional changes outside of the four hypothesized domains likely exist in the offspring’s brain associated with maternal adiposity. In general, the regions detected in the current study showing either localized or global functional connectivity alterations associated with maternal adiposity are largely consistent with a previous study examining structural brain alterations associated with obesity (Shott et al., 2015). Specifically, they reported that obesity is associated with decreases in gray and associated white matter volumes in orbital frontal cortex, insula, amygdala, striatum, hippocampus, and medial prefrontal cortex (Shott et al., 2015). Linking the two sets of findings together, it is possible that the observed functional alterations in neonatal offspring of mothers with high BMI may evolve with age and parallel or precede detectable structural changes later in their life. Therefore, the earlier detection of such functional alterations may provide a new way for early identification of risks and help design potential interventions.

Limitations of this study included the relatively small sample size preventing us from detecting likely more widespread and subtle functional connectivity differences between groups. The existence of below-threshold connectional differences can be partly reflected by the detection of a range of regions showing global degree and/or efficiency changes with or without concurrent observation of local clusters showing connectivity differences. In the future, studies with larger sample sizes are needed to validate and expand the current findings. Another limitation of the current study relates to the lack of follow-up behavioral measures (which are ongoing) in cognitive and/or emotional control domains. With such measures, potential direct links between maternal adiposity-related brain functional alterations and behavioral outcomes could be established. Regarding other covariates, we found significant relationships between gestational weight gain and cluster-wise functional connectivity alterations. This is not surprising since gestational weight gain highly negatively correlated with maternal BMI in the current study (*r* = -0.60, *p* < 0.001). The minimal effects of other potential confounding variables may need to be confirmed in future studies with larger sample sizes. Finally, another limitation of the current study is the potential sleeping stage differences among subjects. Given the practical difficulties of implementing concurrent EEG during MRI scans of naturally sleeping infants, rigorous control of sleeping stage differences in our analysis could not be achieved and future studies are needed to address this issue.

## Conclusion

In conclusion, by comparing whole brain functional connectivity patterns of neonates born to mothers with normal or high BMI, we detected a range of functional connection differences covering all four functional domains of circuits that are critically involved in the etiology of adult obesity. These findings provide important clues to the potential neural basis for cognitive and behavioral differences previously reported for children born to obese mothers. The fact that functional connectivity differences can be observed as early as the neonatal period strongly indicates early-life programming of the neonatal brain in response to maternal obesity. Therefore, future work should concentrate on validating such measures and potentially build novel biomarkers for effective identification of risks and on the design of possible intervention strategies.

## Ethics Statement

This study was carried out in accordance with the recommendations of University of Arkansas for Medical Sciences (UAMS) Institutional Review Board (IRB) with written informed consent from all subjects. All subjects gave written informed consent in accordance with the Declaration of Helsinki. The protocol was approved by the UAMS IRB.

## Author Contributions

AS and WG performed data analysis and wrote part of the manuscript. AA and TB contributed in the study design and edited the manuscript. CG, RR, and AR contributed in data analysis and reviewed the manuscript. XO designed the study, performed data acquisition and some of the analysis, and wrote part of the manuscript.

## Conflict of Interest Statement

The authors declare that the research was conducted in the absence of any commercial or financial relationships that could be construed as a potential conflict of interest.
