# Supplementary material for: Maternal Adiposity Influences Neonatal Brain Functional Connectivity
Source: Front Hum Neurosci. 2019 Jan 4;12:514. doi: 10.3389/fnhum.2018.00514 (PMC6328446; doi:10.3389/fnhum.2018.00514)
Supplement: Supplementary file 1 [file Table_1.DOCX]

Supplement A: AAL abbreviations and functional parcellation sub-region correspondence.

| **Region** | | **Hemisphere** | **Abbreviation** | **funPar #** |
| --- | --- | --- | --- | --- |
| Precentral gyrus |  | left | PreCG-L | 1-2 |
| Precentral gyrus |  | right | PreCG-R | 3-5 |
| Superior frontal gyrus | (dorsal) | left | SFGdor-L | 6-15 |
| Superior frontal gyrus | (dorsal) | right | SFGdor-R | 16-23 |
| Orbitofrontal cortex | (superior) | left | ORBsup-L | 24-27 |
| Orbitofrontal cortex | (superior) | right | ORBsup-R | 28-32 |
| Middle frontal gyrus |  | left | MFG-L | 33-34 |
| Middle frontal gyrus |  | right | MFG-R | 35-38 |
| Orbitofrontal cortex | (middle) | left | ORBmid-L | 39-41 |
| Orbitofrontal cortex | (middle) | right | ORBmid-R | 42-44 |
| Inferior frontal gyrus | (opercular) | left | IFGoperc-L | 45 |
| Inferior frontal gyrus | (opercular) | right | IFGoperc-R | 46 |
| Inferior frontal gyrus | (triangular) | left | IFGtriang-L | 47 |
| Inferior frontal gyrus | (triangular) | right | IFGtriang-R | 48-49 |
| Orbitofrontal cortex | (inferior) | left | ORBinf-L | 50 |
| Orbitofrontal cortex | (inferior) | right | ORBinf-R | 51-52 |
| Rolandic operculum |  | left | ROL-L | 53 |
| Rolandic operculum |  | right | ROL-R | 54 |
| Supplementary motor area |  | left | SMA-L | 55-56 |
| Supplementary motor area |  | right | SMA-R | 57-58 |
| Olfactory |  | left | OLF-L | 59 |
| Olfactory |  | right | OLF-R | 60-61 |
| Superior frontal gyrus | (medial) | left | SFGmed-L | 62 |
| Superior frontal gyrus | (medial) | right | SFGmed-R | 63-67 |
| Orbitofrontal cortex | (medial) | left | ORBmed-L | 68 |
| Orbitofrontal cortex | (medial) | right | ORBmed-R | 69-71 |
| Rectus gyrus |  | left | REC-L | 72-77 |
| Rectus gyrus |  | right | REC-R | 78-83 |
| Insula |  | left | INS-L | 84-85 |
| Insula |  | right | INS-R | 86-87 |
| Anterior cingulate gyrus |  | left | ACG-L | 88-89 |
| Anterior cingulate gyrus |  | right | ACG-R | 90 |
| Middle cingulate gyrus |  | left | MCG-L | 91-92 |
| Middle cingulate gyrus |  | right | MCG-R | 93-96 |
| Posterior cingulate gyrus |  | left | PCG-L | 97 |
| Posterior cingulate gyrus |  | right | PCG-R | 98 |
| Hippocampus |  | left | HIP-L | 99 |
| Hippocampus |  | right | HIP-R | 100-101 |
| ParaHippocampal gyrus |  | left | PHG-L | 102-105 |
| ParaHippocampal gyrus |  | right | PHG-R | 106-107 |
| Amygdala |  | left | AMYG-L | 108 |
| Amygdala |  | right | AMYG-R | 109 |
| Calcarine cortex |  | left | CAL-L | 110 |
| Calcarine cortex |  | right | CAL-R | 111-113 |
| Cuneus |  | left | CUN-L | 114-115 |
| Cuneus |  | right | CUN-R | 116-117 |
| Lingual gyrus |  | left | LING-L | 118-119 |
| Lingual gyrus |  | right | LING-R | 120-121 |
| Superior occipital gyrus |  | left | SOG-L | 122-123 |
| Superior occipital gyrus |  | right | SOG-R | 124-125 |
| Middle occipital gyrus |  | left | MOG-L | 126-127 |
| Middle occipital gyrus |  | right | MOG-R | 128-129 |
| Inferior occipital gyrus |  | left | IOG-L | 130 |
| Inferior occipital gyrus |  | right | IOG-R | 131 |
| Fusiform gyrus |  | left | FFG-L | 132-138 |
| Fusiform gyrus |  | right | FFG-R | 139-144 |
| Postcentral gyrus |  | left | PoCG-L | 145 |
| Postcentral gyrus |  | right | PoCG-R | 146 |
| Superior parietal gyrus |  | left | SPG-L | 147-148 |
| Superior parieta lgyrus |  | right | SPG-R | 149-150 |
| Inferior parietal lobule |  | left | IPL-L | 151-154 |
| Inferior parietal lobule |  | right | IPL-R | 155-156 |
| Supramarginal gyrus |  | left | SMG-L | 157-159 |
| Supramarginal gyrus |  | right | SMG-R | 160 |
| Angular gyrus |  | left | ANG-L | 161 |
| Angular gyrus |  | right | ANG-R | 162-164 |
| Precuneus |  | left | PCUN-L | 165-166 |
| Precuneus |  | right | PCUN-R | 167-168 |
| Paracentral lobule |  | left | PCL-L | 169 |
| Paracentral lobule |  | right | PCL-R | 170 |
| Caudate |  | left | CAU-L | 171-172 |
| Caudate |  | right | CAU-R | 173-175 |
| Putamen |  | left | PUT-L | 176 |
| Putamen |  | right | PUT-R | 177 |
| Pallidum |  | left | PAL-L | 178 |
| Pallidum |  | right | PAL-R | 179 |
| Thalamus |  | left | THA-L | 180 |
| Thalamus |  | right | THA-R | 181-182 |
| Heschl gyrus |  | left | HES-L | 183-184 |
| Heschl gyrus |  | right | HES-R | 185 |
| Superior temporal gyrus |  | left | STG-L | 186 |
| Superior temporal gyrus |  | right | STG-R | 187-193 |
| Temporal pole | (superior) | left | TPOsup-L | 194-197 |
| Temporal pole | (superior) | right | TPOsup-R | 198-202 |
| Middle temporal gyrus |  | left | MTG-L | 203-204 |
| Middle temporal gyrus |  | right | MTG-R | 205-206 |
| Temporal pole | (middle) | left | TPOmid-L | 207-209 |
| Temporal pole | (middle) | right | TPOmid-R | 210 |
| Inferior temporal gyrus |  | left | ITG-L | 211-215 |
| Inferior temporal gyrus |  | right | ITG-R | 216-223 |

Supplement B: Covariate analysis for seed: cluster (top), node degree centrality (middle), and node efficiency (bottom).

| **Seed** | | **FCz ~ Covariate (P-values)** | | | | | | | | | | |
| --- | --- | --- | --- | --- | --- | --- | --- | --- | --- | --- | --- | --- |
| **funPar #** | **AAL** | **Birth Weight** | **Birth Length** | **Gest. Age** | **Age at MRI** | **MIQ** | **Weight 2wk** | **Height 2wk** | **Head Circ. 2wk** | **Gest. weight gain*** | **Childs Gender** | **Childs Race** |
| 1 | PreCG-L | 0.231 | 0.972 | 0.304 | 0.867 | 0.619 | 0.189 | 0.371 | 0.299 | 0.172 | 0.647 | 1.000 |
| 14 | SFGdor-L | 0.070 | 0.886 | 0.881 | 0.117 | 0.428 | 0.134 | 0.521 | 0.269 | **0.010** | 0.748 | 0.366 |
| 41 | ORBmid-L | 0.519 | 0.337 | 0.767 | 0.541 | 0.118 | 0.218 | 0.872 | 0.205 | **0.010** | 0.360 | 0.932 |
| 41 | ORBmid-L | 0.755 | 0.908 | 0.576 | 0.424 | 0.865 | 0.604 | 0.621 | 0.518 | **0.024** | 0.812 | 0.575 |
| 42 | ORBmid-R | 0.442 | 0.491 | 0.749 | 0.082 | 0.415 | 0.535 | 0.928 | 0.209 | **0.009** | 0.536 | 0.415 |
| 66 | SFGmed-L | 0.056 | 0.771 | 0.644 | 0.118 | 0.602 | 0.155 | 0.638 | 0.946 | **0.011** | 0.824 | 0.159 |
| 72 | REC-L | 0.342 | 0.512 | 0.541 | 0.492 | 0.673 | 0.330 | 0.888 | 0.596 | **0.012** | 0.435 | 0.743 |
| 106 | PHG-R | 0.102 | 0.300 | 0.713 | 0.206 | 0.292 | 0.172 | 0.081 | 0.304 | 0.196 | 0.329 | 0.865 |
| 128 | MOG-R | 0.512 | 0.498 | 0.667 | 0.541 | 0.429 | 0.579 | 0.294 | 0.726 | **0.002** | 0.374 | 0.655 |
| 129 | MOG-R | 0.626 | 0.739 | 0.589 | 0.385 | 0.695 | 0.949 | 0.807 | 0.517 | **0.012** | 0.551 | 0.390 |
| 139 | FFG-R | 0.552 | 0.517 | 0.405 | 0.825 | 0.536 | 0.918 | 0.513 | 0.975 | **0.045** | 0.348 | 0.785 |
| 146 | PoCG-R | 0.228 | 0.985 | 0.264 | 0.552 | 0.243 | 0.234 | 0.635 | 0.153 | 0.071 | 0.611 | 0.970 |
| 174 | CAU-R | 0.130 | 0.189 | 0.613 | 0.235 | 0.791 | 0.054 | 0.191 | 0.758 | 0.052 | 0.995 | 0.070 |
| 192 | STG-R | 0.180 | 0.708 | 0.328 | 0.829 | 0.426 | 0.231 | 0.232 | 0.795 | **0.014** | 0.446 | 0.402 |
| 207 | TPOmid-L | 0.175 | 0.857 | 0.834 | 0.582 | 0.852 | 0.474 | 0.956 | 0.449 | **0.047** | 0.563 | **0.019** |
| 207 | TPOmid-L | **0.024** | 0.457 | 0.271 | 0.637 | 0.770 | **0.029** | 0.289 | 0.400 | **0.002** | 0.855 | 0.283 |
| 212 | ITG-L | 0.080 | 0.159 | 0.510 | 0.732 | 0.444 | 0.108 | 0.358 | 0.197 | 0.554 | 0.572 | 0.324 |
|  | | | | | | | | | | | | |
| **Node** | | **DC ~ Covariate (P-values)** | | | | | | | | | | |
| **funPar #** | **AAL** | **Birth Weight** | **Birth Length** | **Gest. Age** | **Age at MRI** | **MIQ** | **Weight 2wk** | **Height 2wk** | **Head Circ. 2wk** | **Gest. weight gain*** | **Childs Gender** | **Childs Race** |
| 14 | SFGdor-L | 0.931 | 0.474 | 0.164 | 0.883 | 0.381 | 0.895 | 0.502 | 0.340 | 0.215 | 0.862 | 0.147 |
| 19 | SFGdor-R | 0.174 | 0.359 | 0.062 | 0.770 | 0.606 | 0.083 | 0.324 | 0.420 | 0.420 | 0.671 | 0.065 |
| 39 | ORBmid-L | 0.473 | 0.390 | 0.974 | 0.368 | 0.870 | 0.503 | 0.102 | 0.426 | 0.219 | 0.752 | 0.940 |
| 41 | ORBmid-L | 0.916 | 0.928 | 0.473 | 0.175 | 0.993 | 0.949 | 0.269 | 0.546 | 0.226 | 0.989 | 0.606 |
| 42 | ORBmid-R | 0.498 | 0.616 | 0.287 | 0.465 | 0.141 | 0.310 | 0.996 | 0.329 | 0.317 | 0.815 | 0.367 |
| 44 | ORBmid-R | 0.394 | 0.237 | 0.688 | 0.858 | 0.800 | 0.081 | 0.357 | 0.056 | 0.366 | **0.012** | 0.199 |
| 62 | SFGmed-L | 0.329 | 0.437 | 0.743 | 0.803 | 0.720 | 0.120 | 0.264 | 0.133 | 0.405 | 0.655 | 0.682 |
| 79 | REC-R | 0.089 | 0.098 | 0.292 | 0.339 | 0.184 | 0.222 | 0.120 | 0.664 | 0.095 | 0.706 | 0.411 |
| 92 | MCG-L | **0.007** | 0.196 | 0.101 | 0.751 | 0.603 | **0.012** | **0.009** | 0.260 | 0.933 | 0.469 | 0.556 |
| 95 | MCG-R | 0.296 | 0.680 | 0.801 | 0.166 | 0.463 | 0.208 | 0.147 | 0.407 | 0.922 | 0.855 | 0.862 |
| 109 | AMYG-R | 0.991 | 0.437 | 0.638 | 0.686 | 0.660 | 0.932 | 0.904 | 0.592 | 0.144 | 0.698 | 0.288 |
| 188 | STG-R | 0.910 | 0.636 | 0.807 | 0.494 | 0.074 | 0.564 | 0.256 | 0.299 | 0.259 | 0.509 | 0.301 |
| 190 | STG-R | 0.447 | 0.161 | 0.681 | 0.463 | 0.893 | 0.317 | **0.048** | 0.715 | 0.871 | 0.182 | 0.996 |
|  |  |  |  |  |  |  |  |  |  |  |  |  |
| **Node** | | **NE ~ Covariate (P-values)** | | | | | | | | | | |
| **funPar #** | **AAL** | **Birth Weight** | **Birth Length** | **Gest. Age** | **Age at MRI** | **MIQ** | **Weight 2wk** | **Height 2wk** | **Head Circ. 2wk** | **Gest. weight gain*** | **Childs Gender** | **Childs Race** |
| 14 | SFGdor-L | 0.966 | 0.466 | 0.157 | 0.995 | 0.706 | 0.838 | 0.446 | 0.261 | 0.192 | 0.969 | 0.297 |
| 19 | SFGdor-R | 0.119 | 0.289 | 0.063 | 0.689 | 0.413 | **0.041** | 0.247 | 0.243 | 0.319 | 0.532 | **0.033** |
| 39 | ORBmid-L | 0.651 | 0.557 | 0.768 | 0.235 | 0.571 | 0.583 | 0.157 | 0.354 | 0.186 | 0.655 | 0.868 |
| 41 | ORBmid-L | 0.781 | 0.702 | 0.677 | 0.105 | 0.797 | 0.945 | 0.422 | 0.637 | 0.125 | 0.825 | 0.523 |
| 42 | ORBmid-R | 0.485 | 0.695 | 0.363 | 0.352 | 0.116 | 0.315 | 0.996 | 0.318 | 0.168 | 0.875 | 0.443 |
| 44 | ORBmid-R | 0.439 | 0.350 | 0.708 | 0.921 | 0.606 | 0.096 | 0.494 | 0.059 | 0.295 | **0.019** | 0.227 |
| 62 | SFGmed-L | 0.417 | 0.651 | 0.914 | 0.965 | 0.879 | 0.144 | 0.338 | 0.106 | 0.203 | 0.688 | 0.626 |
| 79 | REC-R | 0.074 | 0.089 | 0.327 | 0.420 | 0.222 | 0.201 | 0.114 | 0.707 | **0.034** | 0.674 | 0.208 |
| 92 | MCG-L | 0.115 | 0.055 | 0.212 | 0.498 | 0.702 | 0.130 | 0.076 | 0.720 | 0.460 | 0.563 | 0.554 |
| 95 | MCG-R | **0.016** | 0.229 | 0.082 | 0.605 | 0.444 | **0.031** | **0.022** | 0.517 | 0.782 | 0.749 | 0.557 |
| 109 | AMYG-R | 0.343 | 0.604 | 0.553 | 0.340 | 0.302 | 0.293 | 0.185 | 0.648 | 0.787 | 0.627 | 0.874 |
| 188 | STG-R | **0.041** | 0.289 | 0.052 | 0.797 | 0.115 | 0.126 | 0.134 | 0.407 | 0.661 | 0.347 | 0.286 |
| 190 | STG-R | 0.976 | 0.376 | 0.582 | 0.741 | 0.840 | 0.985 | 0.757 | 0.438 | 0.262 | 0.515 | 0.297 |
| 191 | STG-R | 0.924 | 0.763 | 0.675 | 0.350 | 0.162 | 0.674 | 0.324 | 0.284 | 0.226 | 0.501 | 0.264 |
| 192 | STG-R | 0.496 | 0.211 | 0.493 | 0.440 | 0.803 | 0.364 | 0.065 | 0.721 | 0.740 | 0.146 | 0.941 |
